# The effect of acute intermittent hypoxia on postprandial triglyceride levels in humans: a randomized crossover trial

**DOI:** 10.1186/s12967-021-02933-z

**Published:** 2021-06-22

**Authors:** Renée Morin, Jean-François Mauger, Ruwan Amaratunga, Pascal Imbeault

**Affiliations:** 1 School of Human Kinetics, Faculty of Health Sciences, University of Ottawa, Ottawa, ON Canada; 2 Institut du Savoir Montfort, Hôpital Montfort, Ottawa, ON Canada; 3 Behavioural and Metabolic Research Unit, School of Human Kinetics, Faculty of Health Sciences, University of Ottawa, 200 Lees, Ottawa, ON K1N 6N5 Canada

**Keywords:** Triglyceride-rich lipoproteins, Normobaric hypoxia, Obstructive sleep apnea, Triglyceride levels, Intermittent hypoxia

## Abstract

**Background:**

Obstructive sleep apnea (OSA), a sleep disorder frequently observed in individuals living with obesity, consists of repeated involuntary breathing obstructions during sleep, leading to intermittent hypoxia (IH). In humans, acute continuous hypoxia slightly increases plasma triglycerides (TG). However, no study yet compared the postprandial TG response of individuals with or without OSA under intermittent hypoxia.

**Methods:**

Using a randomized crossover design, seven individuals diagnosed with moderate OSA and eight healthy individuals without OSA were given a meal after which they were exposed for 6 h to normoxia or intermittent hypoxia (e.g., 15 hypoxic events per hour). Blood lipid levels were measured hourly during each session.

**Results:**

Peak postprandial TG concentrations tended to be 22% higher under IH irrespective of group (IH × time interaction, p = 0.068). This trend toward higher total plasma TG was attributable to increased levels of denser TG-rich lipoproteins such as very low-density lipoproteins (VLDL) and chylomicrons (CM) remnants. Irrespective of group, the postprandial TG concentrations in denser TG-rich lipoproteins was 20% higher under IH (IH × time interaction, p = 0.036), although IH had virtually no impact on denser TG-rich lipoprotein concentrations in the OSA group.

**Conclusion:**

Acute intermittent hypoxia tends to negatively affect postprandial TG levels in healthy individuals, which is attributable to an increase in denser TG-carrying lipoprotein levels such as VLDL and CM remnants. This altered postprandial TG response to acute intermittent hypoxia was not observed in individuals with OSA.

## Key points

- Obstructive sleep apnea is a sleep disorder commonly observed in individuals living with obesity that consists of repeated episodes of partial or complete obstructions of the upper airway, leading to intermittent hypoxia.
- Obstructive sleep apnea is associated with elevated circulating triglycerides. The postprandial lipemic response of individuals diagnosed with obstructive sleep apnea under intermittent hypoxic conditions remains unknown.
- Under normoxia, we observed an altered postprandial lipemic response in the obstructive sleep apnea group compared to the control group. However, upon 6h of acute intermittent hypoxia, postprandial lipemia was marginally increased in the control group, but did not further worsen in our sample of individuals with obstructive sleep apnea.

## Background

Obesity is known to be a precursor of obstructive sleep apnea (OSA) due to the inverse relationship between obesity and pharyngeal area. Increase in fat deposition in the pharynx eventually causes decrease in patency of the pharynx leading to upper airway collapse [1]. Even modest weight gain/loss has the potential of predicting OSA severity, a 10% weight gain can predict a 32% increase in the apnea–hypopnea index (AHI) while a 10% weight loss predicted a 26% decrease in AHI [32]. In individuals living with obesity, the prevalence of OSA exceeds 50% [44]. OSA consists of repeated breathing obstructions during sleep, usually caused by the obstruction of the upper airway, inducing breathing difficulties. These events can cause rapid depletion/repletion of blood tissue oxygen content, a phenomenon referred to as intermittent hypoxia (IH) [9]. Aside from causing daytime sleepiness, IH can lead to various health consequences, the most salient of which being up to a twofold increased risk of developing cardiovascular disease (CVD) [14, 30]. A potential explanatory factor linking OSA to increased risk of developing CVD is the disturbing effect of OSA on blood lipid levels, more specifically blood triglyceride (TG) levels. In this regard, individuals with OSA usually display 30% greater TG levels compared to individuals without OSA [29].

Plasma TG concentrations are governed via the production of TG-rich lipoproteins (TRLs) by the liver [as very low-density lipoproteins (VLDL)] and the intestine [as chylomicrons (CM)] as well as the disposal of TRL and their remnants by the periphery and the liver [3, 31]. Considerable research has investigated the effect of acute or chronic moderate hypoxia on circulating TG levels. In rodents, it has been shown that acute hypoxia increases TG levels through two major mechanisms: increased hepatic secretion of VLDL and decreased TRL clearance [18]. This effect of hypoxia, however, was not observed when rodents were kept in thermoneutral conditions [19]. Studies conducted in healthy human volunteers and which controlled for critical confounders likely to alter plasma TG such as physical activity, diet and body weight variation, suggest that acute hypoxia appears to only marginally affect triglyceridemia. We have reported that 6 h of IH at rest did not change postprandial TG levels in healthy individuals [25]. We also showed that an acute 6-h exposure to continuous (i.e., not intermittent) normobaric hypoxia does not impact circulating TG levels in the fasted state [26], but may slightly rise prandial lipemia in the constantly fed state [27]. In individuals with OSA, on the other hand, the effect of chronic exposure to intermittent hypoxia may lead to deleterious alterations in lipid metabolism and lipemia. For instance, under normoxic conditions, Drager et al. observed a pronounced sixfold reduction in daytime clearance rate of plasma triglycerides in individuals with severe OSA compared to healthy individuals [11]. To our knowledge, no study yet directly compared the postprandial lipemic response of individuals diagnosed with OSA to that of healthy individuals under both normoxic and hypoxic conditions. Accordingly, the objective of this study was to compare the effect of an acute 6-h exposure to intermittent hypoxia on the postprandial TG content of buoyant and denser TG-rich lipoproteins in humans with and without moderate OSA. Based on the facts that individuals with OSA may already be impaired in terms of lipid metabolism and may also chronically display higher than normal sympathetic tone compared to healthy individuals [40], we hypothesized that individuals with OSA would display an altered postprandial lipemic response compared to healthy individuals and that IH would further worsen postprandial lipemia in this group.

## Materials and methods

### Participants

Our sample was composed of seven individuals diagnosed with moderate sleep apnea (aged 44–65) recruited from the Sleep Laboratory at Montfort Hospital as well as 8 healthy individuals (aged 21–24) without OSA from the University of Ottawa. Participants provided written consent and the study protocol was approved by the Research Ethics Board of the University of Ottawa and from Montfort Hospital. Exclusion criteria included: history of physician-diagnosed asthma or other respiratory illness, hypertension, cardiovascular diseases, diabetes, habitual sleep duration of less than 7 h per night, allergies to lactose, current smoking status, and current and regular use of statins and/or fibrates known to have an impact on lipid metabolism.

### Anthropometric and metabolic measurements

Body weight and height were measured using a standard beam scale (HR-100, BWB-800AS; Tanita, Arlington Heights, IL) and a standard stadiometer (Perspective Entreprises, Portage, Michigan, USA) respectively. Body composition was measured via percentage of fat mass (%FM), total fat mass (FM), and fat-free mass (FFM) using dual energy X-ray (DXA) (General Electric Lunar Prodigy, Madison, Wisconsin; software version 6.10.019). Resting energy expenditure (REE) was measured in a thermoneutral dark room for 30 min using a Vmax Encore 29 System metabolic cart (VIASYS Healthcare inc, Yorba Linda, CA) following a 12 h overnight fast. Daily energy expenditure (DEE) on experiment days was calculated by multiplying the participants REE by a physical activity factor of 1.375 [16].

### Experimental protocol and procedures

This study was a randomized crossover study consisting of two experimental sessions. Before each experimental session, volunteers were expected to follow pre-experimental criteria; 7 h of sleep per night, restrain from moderate-intense physical activity, caffeine, and alcohol for 36 h, and to consume a provided standardized evening dinner between 7:00 and 8:00 PM (650 kcal; 54% from carbohydrates, 22% from fat, and 24% from protein). Each participant underwent three sessions; one preliminary session and two experimental sessions. During the preliminary session, the participant was given an in-depth explanation of the protocol and the experimental sessions. Informed consent was then collected. The preliminary session also consisted of taking anthropometric measurements (height, weight and body composition). A dual-energy X-ray absorptiometry (DXA) scan was performed to quantify FM and FFM. We then continued by measuring REE by indirect calorimetry. Standard procedures such as 12 h fast, thermoneutral room, and supine posture were followed for both tests. The participant was then exposed to hypoxia for 20 min [by using our experimental setup and exposing the participant to medical nitrogen (N_2_)] in order to detect any symptoms related to altitude sickness (i.e., headache, nausea, dizziness). When exposing the participant to IH cycles, he or she was resting in a reclining seated position. A participant was considered tolerant to hypoxia if no symptoms occurred (dizziness, nausea, extreme fatigue). The researcher then reminded the participant of pre-experimental session criteria and provided the participant with the standardized meal to be consumed the night before the first experimental session. On experimental session days, the participants arrived between 6 and 8 AM following a 12 h overnight fast and they completed a questionnaire that ensured that all the pre-experiment criteria had been met. Both experimental sessions had the same environmental conditions (temperature and humidity) and the study was done in thermoneutrality in order to avoid differences in effect on lipid metabolism [19]. Body mass was measured and compared with measurements taken during the preliminary session. A phlebotomy-certified researcher or nurse collected a 20-ml blood sample after inserting an IV catheter in the forearm. After the baseline blood sample was taken, participants consumed a liquid fat-rich meal (60% fat), representing 33% of the DEE (obtained by indirect calorimetry at rest during a preliminary session multiplied by a physical activity factor of 1.375 [16]) and were then exposed to either intermittent hypoxia or to ambient air (normoxia for 6 h. Blood was drawn every 30 min of the experimental session for the first 2 h and every hour for the reminder of the session; resulting in a total of nine samples (9 × 20 ml = 180 ml. Volunteers remained in a semirecumbent position, and occupied themselves by watching television or reading a book; sleeping was not allowed. In order to induce the simulation of IH, participants wore an oro-nasal mask with a two-way Hans Rudolph non-rebreathing valve that was connected to an inspiratory line [23]. In the normoxia condition, participants only inspired ambient air (20.93% oxygen). In the IH condition, pure medical nitrogen was inspired by the participant through the inspiratory line. Participants inspired nitrogen until oxyhemoglobin saturation (S_p_O_2_) dropped to at least 85%. Once oxyhemoglobin saturation reached 85%, N_2_ flow was stopped, and participants proceeded to inspire ambient air containing oxygen. Using this protocol, an average of 15.5 (3.8) hypoxic events could be induced per hour (simulating moderate OSA).

None of our participants failed the 20 min hypoxia tolerance test in the preliminary session and no participants dropped out due to symptoms related to intermittent hypoxia during the experimental sessions. No validated scale or questionnaire were used to monitor altitude sickness symptoms. Participants were instead asked frequently to report any discomfort related to altitude sickness with special attention to symptoms listed in the Lake Louise consensus scoring system: headache, gastrointestinal upset (anorexia, nausea, or vomiting), fatigue or weakness, and dizziness/light-headedness [38].

### Clinical relevance of IH exposure in the postprandial state

Postprandial hypertriglyceridemia is linked to an increased risk of CVD and mortality while fasting TG levels are not directly atherogenic [10]. Considering that the postprandial increase in triglyceridemia occurs for up to 7–8 h and that studies have reported a peak in plasma TG at approximately 3:00 A.M. following a meal before bedtime [34], it appears realistic to think that a significant amount of the time spent asleep at night occurs in the postprandial state and that is clinically relevant to examine the effect of IH exposure in a postprandial state. Additionally, it is interesting to mention that the circadian cycle influences postprandial lipid metabolism so that a meal eaten at night leads to higher TG concentrations than a meal eaten during the day [21]. This last observation could be of clinical importance in the case of individuals who frequently consume late night snacks. For these reasons, we believe that it is important to better understand how IH affect postprandial lipid metabolism in order to elucidate the mechanisms by which OSA increases CVD risk.

Oxyhemoglobin saturation (S_p_O_2_) and heart rate (HR) were monitored by pulse oximetry and recorded in 5 s intervals during both the normoxia and hypoxia sessions using a Masimo radical 7 unit (Masimo, Irvine, Ca, USA). Complete S_p_O_2_ and HR time series were available for only seven participants (3 OSA and 4 CTL). A mean average was calculated with all the values for each experimental session. Blood pressure (BP) was also measured at baseline and every 30 min of the experimental session for the first two hours and every hour for the reminder of the session (T0, T30, T60, T90, T120, T180, T240, T300, and T360) with an automatic sphygmomanometer (American Diagnostic Corporation, E-sphyg 2, Hauppage NY, USA) following the Canadian Society of Exercise Physiology (CSEP) standard procedures [6].

### Plasma analyses

Plasma was separated immediately after blood collection by centrifugation at 3000 rpm for 10 min at 4 °C. Plasma total TG concentrations were measured by a colorimetric enzymatic assay (Wako Diagnostics). Plasma levels of glucose, insulin and non-esterified fatty acids (NEFA) were measured at baseline (pre-test meal) and 60, 90, 120, 180, 240, 300, and 360 min after test meal ingestion. Concentrations were assayed using enzymatic spectrophotometric analysis (glucose and NEFA) or commercially available enzyme-linked immunosorbent assay (ELISA) kits (insulin), as previously described [17]. Denser lipoprotein TG concentration was calculated by subtracting CM TG concentrations from total plasma TG concentrations.

### Buoyant triglyceride-rich lipoproteins isolation

As recently published, for each participant, buoyant triglyceride-rich lipoproteins (TRL), containing mainly chylomicrons, were isolated using a modified method from Calsake and Packard [37]. Briefly, 1.5 ml of prandial plasma was overlayed with 0.5 ml of sterile saline and centrifuged for 40 min at 12,000 rpm (20,000 RCF at maximal radius) at 4 °C using a Thermo Fisher Scientific 30 × 2 fixed-angle sealed rotor (75003652) in a Sorvall ST16R centrifuge. Infranatant (1 ml) was discarded, 0.5 ml of sterile physiological saline was added to the supernatant and the procedure was repeated two times for a total of three centrifugations. After the final centrifugation, 1.5 ml of infranatant was removed so that the final volume of the buoyant TRL extraction was 0.5 ml.

### Statistical analysis

**Fig. 1:**
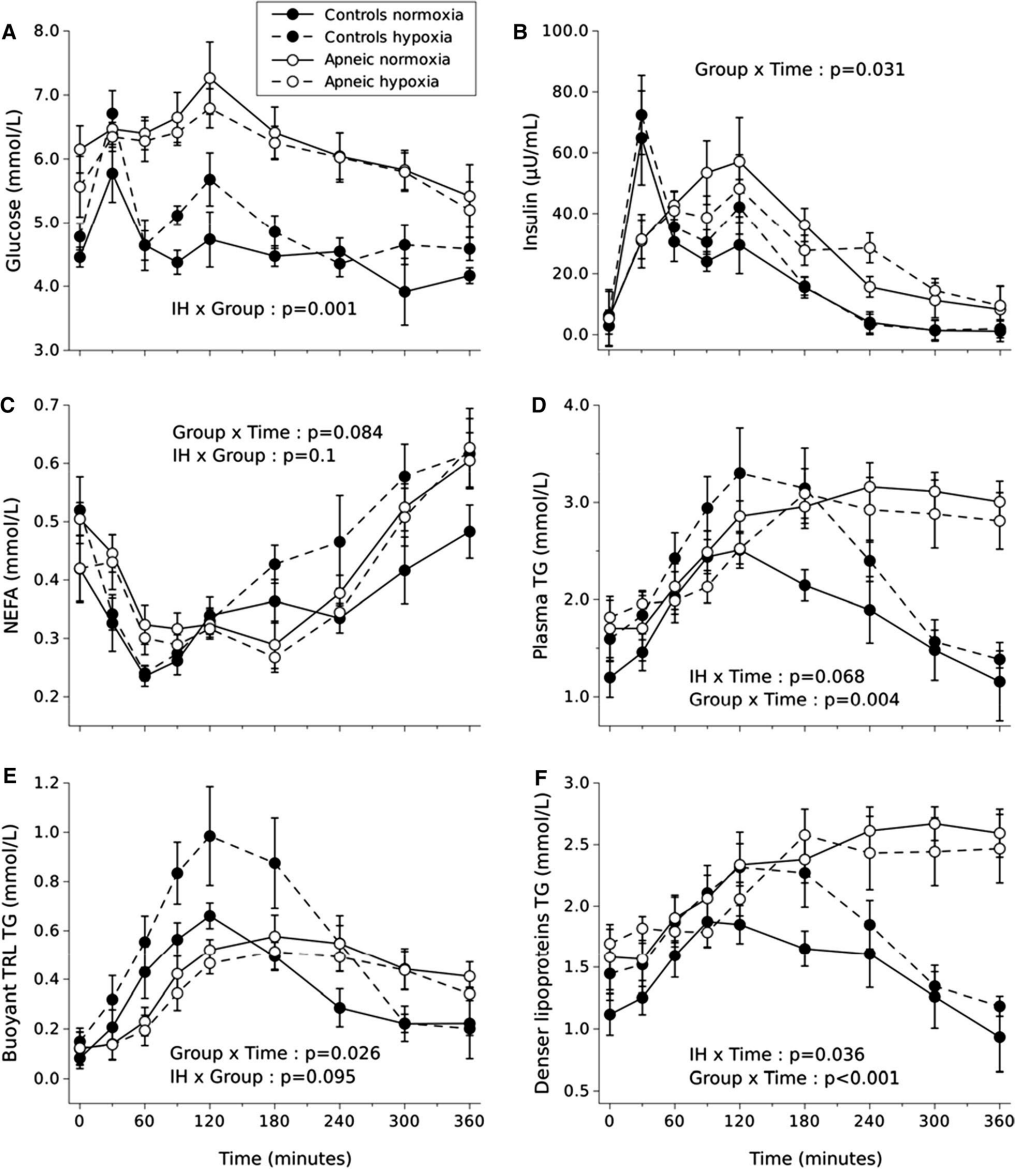
Fasting and postprandial plasma glucose (**A**), insulin (**B**), NEFA (**C**), total TG (**D**), buoyant TRL TG (**E**), and denser lipoproteins TG (**F**) levels measured during normoxia and IH in a group of individuals with moderate OSA (apneic) vs. healthy controls. Values are means ± standard errors adjusted for between- subject’s variability. Main effect of time, p < 0.05 for glucose, insulin, NEFA, total TG, buoyant TRL TG, and denser lipoproteins TG

Data were analyzed using a two-way repeated measure analysis of variance (ANOVA) with time and condition (normoxia vs IH) as within-subject’s parameters and group (i.e., OSA vs control) as a between-subject factor. Identification of a significant interaction led to further analysis of a simple main effects for group or IH. The Greenhouse–Geisser correction was used whenever the sphericity assumption was violated. Partial eta squared (ηp2) was used to estimate the proportion of the variance attributed to the tested factor. Error bars in Fig. 1A–F. were adjusted to eliminate between subjects’ variability and better reflect the statistical power of the study crossover design (6). Statistical analyses were conducted using the Jamovi statistical software (Version 0.9^1^, **RRID:SCR_016142**). Alpha was set at 0.05 to establish significance.

## Results

### Characteristics of participants

Participant characteristics and fasting metabolic plasma parameters are summarized in Tables1, 2. respectively. Participants from both groups remained weight stable (average absolute weight difference between experimental sessions: 0.38 kg OSA and 0.61 kg control) during the experiment. Experimental sessions were separated by 9 (3) days on average for the OSA group and 14 (7) days on average for the control group. Age and fat mass (%) were significantly different between groups (Table 1). Baseline fasting metabolic parameters were very similar between the two experimental sessions in both groups with only glucose being statistically but marginally different between hypoxia and normoxia in the control group, p < 0.05.

**Table 1:** Characteristics of participants (OSA group, n = 7; control group, n = 8)

Characteristics of participants	OSA group (men: N = 6 women: N = 1)	Control group (men: N = 8)	P values
Age (years)	54.4 (6.4)	22.0 (1.1)*	< 0.01
Height (cm)	174.8 (5.8)	183.0 (8.8)	0.057
Weight (kg)	90.5 (22.6)	86.6 (11.2)	0.630
BMI (kg/m^2^)	29.4 (6.6)	25.8 (4.8)	0.277
Lean mass (kg)	57.7 (12.4)	64.9 (2.8)	0.109
Fat mass (kg)	30.2 (14.6)	19.1 (11.7)	0.088
Fat mass (%)	33.0 (8.7)	23.0 (10.1)*	0.021

Data are expressed as mean (standard deviation)

*Significantly different compared to OSA group, p < 0.05

**Table 2:** Fasting metabolic plasma parameters

	OSA		Control	
	Normoxia	Hypoxia	Normoxia	Hypoxia
Triglyceride (mmol/L)	1.7 (0.9)	1.8 (0.5)	1.2 (0.6)	1.6 (0.6)
Non-esterified fatty acids (mmol/L)	0.5 (0.1)	0.4 (0.1)	0.4 (0.2)	0.5 (0.2)
Glucose (mmol/L)	6.2 (1.0)	5.6 (1.3)	4.5 (0.4)	4.8 (0.6)*
Insulin (μU/mL)	5.5 (24.7)	5.3 (23.4)	2.9 (7.6)	6.4 (4.8)

Data are expressed as mean (standard deviation)

*Significantly different compared to normoxia in the control group, p < 0.05

### Cardiorespiratory responses to intermittent hypoxia

Table 3 shows the variations in HR, blood pressure and oxyhemoglobin saturation during both experimental sessions. During IH, an average of 15.5 (4.8) hypoxic cycles were induced per hour, simulating moderate OSA. IH led to a significant increase in mean HR in the control group only.

**Table 3:** Mean hypoxia counts, blood pressure, heart rate oxyhemoglobin saturation during normoxia and intermittent hypoxia in the OSA and control group

		OSA		Control	
		Normoxia	Hypoxia	Normoxia	Hypoxia
Frequency/hour		0.0 (0.0)	15.0 (3.8)	0.0 (0.0)	16.0 (5.7)
Systolic blood pressure (mmHg)		135.0 (19.4)	135.0 (19.4)	124.6 (4.0)	128.4 (8.6)
Diastolic blood pressure (mmHg)		79.4 (9.75.)	79.4 (8.7)	65.3 (6.1)	67.6 (5.4)
Heart rate (BPM)					
Mean		78.7 (11.0)	79.6 (7.0)	61.8 (14.1)	68.3 (14.9)*
Maximum		118.3 (2.31)	98.7 (17.2)	97.8 (11.5)	102.5 (9.6)†
S_p_O_2_ (%)					
Mean		94.1 (2.2)	91.9 (1.7)†	96.8 (0.4)	93.8* (1.1)*
Maximum		98.3 (1.5)	100.0 (0.0)	100.0 (0.0)	99.8 (0.5)
Minimum		82.7 (6.4)	62.0 (7.2)†	92.0 (2.2)	53.3 (4.9)*
Time S_p_O_2_ ≤ 90(%) min		33.2. (55.9)	76.3 (36.0)	0.1 (0.2)	53.2 (22.2)*
Time S_p_O_2_ ≤ 85(%) min		0.5 (0.5)	26.3 (2.3)†	0.0 (0.0)	26.0 (16.2)*
Time S_p_O_2_ ≤ 80(%) min		0.1 (0.1)	13.5 (3.9)†	0.0 (0.0)	12.7 (7.8)*

Data are expressed as mean (standard deviation)

*Statistically significant difference between normoxia and intermittent hypoxia, p < 0.05

†Statistical trend between normoxia and intermittent hypoxia, p < 0.1

### Plasma glucose and insulinemia

The effects of IH on plasma glucose and insulin concentrations are summarized in Fig. 1A, B. Irrespective of time, mean glucose levels were significantly greater under IH compared to normoxia in the control group while they were significantly lower under IH compared to normoxia in the OSA group (IH × group interaction, p = 0.001, η_p_^2^ = 0.585). In both conditions, insulinemia’s peak occurred later in the OSA group and remained higher over time as compared to control (group × time interaction, p = 0.031, η_p_^2^ = 0.219). Irrespective of time, IH tended to increase insulinemia (+ 20%) in the control group and decrease insulinemia in the OSA group (− 6%) (IH × group interaction, p = 0.084, η_p_^2^ = 0.213).

### Plasma lipid levels

Postprandial plasma NEFA, TG, chylomicron TG and denser lipoprotein TG levels during normoxia and intermittent hypoxia sessions are shown in Fig. 1C–F.

As shown in Fig. 1C, NEFA levels seemed to be affected by IH over time only in the CTL group (IH × group × time, p = 0.145, η_p_^2^ = 0.130). Intermittent hypoxia tended to induce a greater increase in NEFA concentrations in the control group (IH x time, p = 0.058, η_p_^2^ = 0.256). Average NEFA concentrations tended to be 24% higher in the CTL group under IH compared to normoxia while IH had virtually no impact on plasma NEFA concentrations in the OSA group (IH × group, p = 0.100, η_p_^2^ = 0.209).

Postprandial plasma TG levels (Fig. 1D) evolved in a significantly different manner across time between groups (group × time interaction, p = 0.004, η_p_^2^ = 0.405). Specifically, postprandial total plasma TG levels failed to return to baseline concentrations 6 h post meal ingestion in the OSA group in both conditions. Postprandial TG concentrations tended to be 22% higher under IH irrespective of group (IH × time interaction, p = 0.068, η_p_^2^ = 0.155), an observation mainly driven by a 21% increase in peak postprandial triglyceridemia under IH in the CTL group.

*The TG levels, discussed in the following sections, were measured in two lipoprotein subtypes: buoyant TG-rich lipoproteins comprising mostly chylomicrons *(*CM*, *and denser lipoproteins TG comprising CM remnants and VLDL*)*.*

#### Buoyant TRL-TG

Postprandial buoyant TRL-TG (Fig. 1E) also evolved in a significantly different manner across time between groups irrespective of the condition (group × time interaction, p = 0.026, η_p_^2^ = 0.232) with buoyant TRL-TG mainly failing to return to baseline levels after 6 h in the OSA group. Buoyant TRL-TG tended to be increased by 35% under IH in the CTL group whereas IH had virtually no effect on buoyant TRL-TG in the OSA group (IH × group, p = 0.095, η_p_^2^ = 0.215).

#### Denser lipoproteins TG

Denser lipoproteins TG evolved differently over time between the control and the OSA group. More specifically, the OSA group’s denser TRL-TG levels were higher than the CTL group’s values across time and failed to return to baseline levels at the end of the experiment (group × time, p = 0.001, η_p_^2^ = 0.414). Irrespective of group, the peak average TG concentrations in denser TRL, which occurred at 180 min (when groups are combined, data not shown), was 20% higher under IH (IH × time, p = 0.036, η_p_^2^ = 0.173), although, as shown in Fig. 1F, IH had virtually no impact on denser TRL-TG concentrations in the OSA group.

## Discussion

In this study, we examined the effect of acute IH on postprandial TG levels in healthy adults and in individuals with OSA. We expected that individuals with OSA would display an altered postprandial lipemic response compared to healthy individuals and that IH would further worsen postprandial lipemia in this group. Our hypothesis was not fully confirmed. Under normoxia, we observed an altered postprandial lipemic response in the OSA group compared to the CTL group. However, upon IH, postprandial lipemia was increased in the CTL group, but did not further worsen in our sample of individuals with OSA. In the CTL group, IH induced a significant progressive increase in plasma NEFA and a transient rise in total plasma TG, the latter mainly attributable to a rise in the TG content of denser TRL fraction composed of chylomicron remnants and VLDL, but also to a rise in buoyant TRL TG. Overall, these results improve our knowledge of the effects of hypoxia on lipid metabolism in healthy individuals and provide new information regarding the homeostasis of lipid metabolism in individuals with OSA.

The IH model we use induces sharp transient increases in HR that are likely explained by a shift in sympathovagal balance toward an increase in sympathetic nervous system activity [23]. Interestingly, the average HR was significantly increased under IH in the CTL group only. The absence of change in mean HR during IH in our OSA subjects, who are older than our participants in the CTL group, is concordant with the blunted cardiac chronotropic function in response to hypoxia previously reported with aging [22].

Due to certain observations that hypoxia may increase basal glucose uptake in human adipocytes and improve insulin sensitivity, it has been argued that hypoxia may provide a potential therapeutic intervention in order to attenuate disturbances in glucose metabolism [42]. Short exposure to IH has been shown to significantly decrease plasma glucose levels in individuals living with obesity while normoxia did not [42]. Interestingly, our study yielded similar results in our group of individuals with OSA upon hypoxia. More specifically, postprandial glycemia decreased significantly under IH for the OSA group while remaining increased in the control group. Furthermore, Mackenzie et al*.* [24] demonstrated that hypoxia significantly decreased plasma insulin levels in type 2 diabetes patents. In this line, our results show that postprandial insulin levels decreased under hypoxia in OSA while they increased in the control group. Using more sophisticated measures of insulin sensitivity to further explore the potential hypoxia exposure, Lecoutre et al. [20] assessed insulin sensitivity through hyperinsulinemic-euglycemic clamp following 10 consecutive nights of moderate hypoxia and found that insulin sensitivity was improved. Strikingly, the improvement in insulin sensitivity was significantly more pronounced in the group of individuals with the lowest baseline insulin sensitivity, suggesting the potential for hypoxia therapy in people with severe insulin resistance. Overall, the effect of IH on lowering glucose and insulin levels in individuals with OSA in our study is interesting and supports the literature reporting the positive effects of hypoxia on improving glycemia and insulinemia in individuals with metabolic complications.

Most recent studies investigating the effects of hypoxia in humans, either continuous or intermittent, have reported that hypoxia induces increases in plasma NEFA [4, 25, 27]. In line with these observations, the current study shows that postprandial NEFA levels increase over time under IH compared to normoxia in the CTL group (IH × time, p = 0.058), but not in the OSA group. This unexpected and interesting divergence of NEFA responses to hypoxia between our two experimental groups is not readily explainable. It is suggested that the increase in circulating NEFA levels commonly observed upon hypoxia could be explained by either: (1) an increase in sympathetic tone, which stimulates adipose tissue lipolysis; (2) an impairment in insulin sensitivity, which impedes the anti-lipolytic action of insulin and/or (3) a decrease in circulating fatty acid utilization by peripheral organs. Which of these mechanisms is or are involved in the robust NEFA-rising effect of hypoxia is still currently unknown. However, the absence of change in postprandial NEFA in the OSA group under moderate IH is likely due to hypoxia not having one or more of these deleterious effects in the present experimental circumstances. Of note, individuals with OSA have higher than normal sympathetic activity both when awake and during sleep, in comparison to healthy individuals [40] and chronic elevation in sympathetic tone has been linked to both insulin resistance [12] and a decreased responsiveness to sympathetic stimulation [33]. These observations could, on the one hand, explain the apparent insulin resistance observed in the OSA group, and on the other hand, explain why insulin sensitivity did not appear to be adversely affected by moderate IH in these individuals. Whether more severe hypoxic conditions are required to induce disturbances in NEFA concentrations in OSA individuals will need to be elucidated and warrant further studies.

Recent studies in humans suggest that the effects of acute hypoxia, either intermittent or continuous, on plasma TG are much less robust than its impact on plasma NEFA [4, 25–27]. We reported that an acute 6-h exposure to IH does not increase postprandial plasma TG in a group of healthy young men similar to our CTL group [25]. However, in this previous study, only total plasma TG were examined whereas in the current study, plasma TG were separated into a buoyant TRL fraction, enriched in intestinal born chylomicrons, and a denser TRL fraction, likely enriched in chylomicron remnants and liver born VLDL. In the present study, the inclusion of the OSA group revealed that acute moderate IH may adversely affect plasma TG metabolism in healthy individuals, but much less so in individuals with OSA. Postprandial total TG levels tended to be higher under IH irrespective of group (IH × time, p = 0.068), this effect being more obvious in the CTL group. The higher postprandial total TG levels trend under IH was mainly attributable to a higher peak in the TG content of denser TRL (IH × time, p = 0.036) and to a trend toward an increase in the TG content of buoyant TRL in the CTL group (IH × group, p = 0.095). It is not clear why we observed changes in postprandial TG concentrations under hypoxia in the present study while such changes were not seen in our previous study using a similar postprandial IH protocol [25]. This divergence could possibly be attributed to the fact that, for unclear reasons, higher average peak postprandial TG concentrations were achieved in the present study (~ 3 mmol/L) compared to the previous one (~ 2 mmol/L). The higher postprandial triglyceridemia achieved in the present study could also be responsible for the fact that we observed a trend toward an increase in the TG content of buoyant TRL, an effect that was not seen in a previous study we conducted in healthy young men in prandial state under continuous hypoxia (in which plasma TG concentrations around 1.8 mmol/L were achieved on average) [27]. This last study allowed us to hypothesize that hypoxia, in healthy individuals, may increase the hepatic production of VLDL-TG in response to an increase in lipid influx in the form of elevated plasma NEFA and chylomicron remnants. Assuming that lipid absorption is not affected by hypoxia, the present study suggests that hypoxia may also impair the clearance of TRL-TG as shown by the accumulation of buoyant TRL TG under IH in the control group. Taken together, recent [27] and current results suggest that alteration in plasma TG under hypoxia in healthy individuals seems to occur only in the prandial and postprandial states and that the intensity of the lipid challenge, measured in terms of peak triglyceridemia, may be a critical factor for these effects to be observed. Our initial hypothesis that individuals with OSA would display an altered postprandial triglyceridemia was confirmed based on the fact that plasma TG were still on average significantly higher in the OSA group 6-h post meal ingestion under normoxia. Our observations are consistent with those of Drager et al*.* [11] who showed that individuals with severe OSA have a markedly diminished peripheral TG clearance rate under normoxic conditions. Our other hypothesis stating that hypoxia would further adversely alter postprandial lipemia in OSA individuals, however was not confirmed, as seen in panel D, E and F of Fig. 1, which clearly suggests that postprandial triglyceridemia was not affected by IH in this group. OSA is recognized to induce chronic sympathetic activation [40] and insulin resistance [36] which could in turn lead to elevated NEFA levels, a key substrate for TRLs production by both the liver and gut [8, 43]. This may explain the absence of deleterious impact of moderate IH on postprandial TG in the OSA group. One may also suggest that our participants with OSA being older may have influenced the divergent effects of IH on postprandial TG observed between our two groups. Aging is indeed associated with a progressive decline in hypoxic sensitivity, as measured by a lower central respiratory drive [13] or a lower cardiac chronotropic function [22]. Another speculative explanation resides in the fact that polymorphisms in genes whose products have an important role in lipid metabolism, such as apoE, TNF-alpha and PPAR-gamma have been linked to an increased risk of developing OSA [28]. Since participants were not screened for such polymorphisms, these associations could have translated into a higher prevalence of lipemia-altering polymorphisms in our OSA group and contributed to its deteriorated postprandial lipemic response under normoxia and blunted its response to hypoxia. It should however be noted that, at least in the case of apoE, the deleterious effect of hypoxia on plasma lipid concentrations have been shown to be stronger than the effect attributed to the presence of polymorphic alleles [41]*.*

There exist several treatment avenues for OSA with continuous positive airway pressure (CPAP) being the most generally prescribed and effective [7]. Various studies have demonstrated that an adequate CPAP treatment regimen attenuates the OSA related increased risk in developing CVD, lowers blood pressure, partially reverses metabolic abnormalities, and improves postprandial lipemia [2, 34, 39]. Interestingly, not only did Phillips et al. [34] demonstrate that CPAP reduces postprandial TG and total cholesterol levels at night, but there was an additional reduction in daytime postmeal TG levels by 0.5 mmol/L with CPAP. These results could imply that the association between OSA and CVD may be caused by direct effects on dyslipidemia [34]. While CPAP is a highly effective treatment for OSA, its main limitation is related to a problem of adherence. Indeed, failure to comply with treatment has been reported to be as high as 25–50% [45]. Knowing this, the importance of further research is crucial and could help in the development of alternative strategies/treatment for OSA.

## Weaknesses and strengths

There are limitations to this study. The main weakness being the size and composition of the cohort. The sample of this study is only composed of seven individuals with OSA and eight individuals without OSA that are not matched for age and body composition, making it difficult to isolate the effect of OSA per se on postprandial lipoprotein metabolism. The sample being principally composed of men also limits the generalization of our findings to the overall population. There also exists some weaknesses regarding our model of simulated OSA. Limitations include the rapid desaturation and resaturation of the obstructive events which are typically slow in real OSA as well as the absence of arousal from sleep and hypercapnia. Additionally, our experimental model is set during daytime while the participants are awake, which is opposite of the hallmark of OSA [35] and does not take into account the impact of the timing of the meal prior to sleep on postprandial lipid metabolism [15]. It is also important to mention that the duration of the hypoxia session, being of 6 h, is of a shorter duration that may occur in undiagnosed and/or non-treated individuals living with OSA. Although our study has a relatively small sample size, we were able to detect significant increases in certain plasma parameters due to the statistical strength of the crossover design. Another strong aspect of our study is the standardized diet of the participants the day before each experimental session in order to obtain comparable fasting metabolic plasma parameters.

## Conclusion

The present work further supports our knowledge of the effects of hypoxia on lipid metabolism in healthy individuals and provides new information regarding the homeostasis of lipid metabolism in individuals with OSA. In normoxia, individuals with OSA displayed an altered postprandial lipemic response compared to the CTL group. Upon IH, postprandial lipemia was marginally increased in the CTL group, with no further detrimental effect in our sample of individuals with OSA. In the CTL group, IH induced a significant progressive increase in plasma NEFA and a transient rise in total plasma TG, the latter mainly attributable to a rise in the TG content of denser TRL fraction composed of chylomicron remnants and VLDL. Our results partially support previous observations from animal and human studies by demonstrating that acute IH, a simulating model of OSA, tends to negatively affect postprandial TG levels, which is attributable to an increase in denser TG carrying lipoprotein levels such as, VLDL and CM remnants. Further studies should be conducted for developing strategies that could alleviate or counter the effects of IH on TRL denser lipoprotein levels and therefore concomitantly reduce the associated cardiovascular risk associated with OSA.

## Data Availability

The datasets generated for this study are available on request to the corresponding author.

## Abbreviations

CVD: Cardiovascular disease
NEFA: Non-esterified fatty acids
OSA: Obstructive sleep apnea
O_2_: Oxygen
N_2_: Nitrogen
TG: Triglycerides
VLDL: Very-low density lipoprotein
CM: Chylomicrons
TRL: Triglyceride-rich lipoprotein
IH: Intermittent hypoxia
AHI: Apnea–hypopnea index
S_p_O_2_: Oxyhemoglobin saturation
HR: Heart rate
CTL: Control
